# Validation of the simplified Chinese (Mainland) version of the Disability of the Arm, Shoulder, and Hand questionnaire (DASH-CHNPLAGH)

**DOI:** 10.1186/s13018-015-0216-6

**Published:** 2015-05-23

**Authors:** Hua Chen, Xinran Ji, Wei Zhang, Yiling Zhang, Lihai Zhang, Peifu Tang

**Affiliations:** Department of Orthopaedic Surgery, The General Hospital of People’s Liberation Army (301 Hospital), 28 Fuxing Road, Wukesong, Beijing, 100000 China

**Keywords:** Disability of the Arm, Shoulder, and Hand (DASH), Simple Chinese version of DASH (DASH-CHNPLAGH), Reliability, Validity

## Abstract

**Background:**

We developed a simplified Chinese version of the Disabilities of the Arm, Shoulder, and Hand (DASH) questionnaire (shorthand for DASH-CHNPLAGH) by conducting cross-cultural adaptation and evaluating its reliability and validity.

**Methods:**

Both the forward and back translations were performed by language officers and medical professionals from different fields to translate the original DASH version into the DASH-CHNPLAGH version with minimized culture discrepancies. A total of 300 patients with upper extremity disorders were recruited to complete two DASH-CHNPLAGH questionnaires with 3-week test interval time.

**Results:**

The intraclass coefficient (ICC) and Cronbach’s alpha for the 30-item disability/symptom of the DASH-CHNPLAGH was 0.94 and 0.96, respectively, which were relatively high among that reported in previous literatures. DASH-CHNPLAGH showed a positive correlation with a visual analogue scale (VAS) and a negative correlation with SF-36 items.

**Conclusion:**

The simple Chinese version of DASH is a reliable and valid measurement for patients with upper extremity problems.

## Background

The main factors affecting the function of the upper extremity are pain, range of motion, and muscle power. Dysfunction related to these problems will negatively affect the health-related life quality [1]. A number of questionnaires for assessment of muscle strength, functional, limitations, range of motion, and quality of life have been developed to analyze the course of the upper extremity disease or the effectiveness of treatment. Although these questionnaires reflect the changes of quality during medical care, not all of them are practically used for analyzing the overall musculoskeletal disabilities [2].

The Disability of the Arm, Shoulder, and Hand (DASH) is a region-specific data collection instrument for measurement of the functional status, different degrees of symptoms, and upper extremity disability levels, which has been developed by the “American Academy of Orthopaedic Surgeons” and “Institute for Work and Health” [3, 4]. Nowadays, the use of the DASH has been rapidly applied in clinical trials and has proven reliability and validity as a self-administered measurement. The psychometric properties of its English and European language versions have been examined [5, 6], and it is now available in other language versions such as Korean [7], Japanese [8], Arabic [9], etc. However, information about DASH measurement properties in the cross-cultural adaptation versions is still inadequate and needs more studies with high methodological qualities for accurate assessment.

To use a questionnaire efficiently in different language groups and cultural background, it must not only be translated into the new language properly but also be adapted to the new culture and be verified if there were culture difference between them. Recently, two Chinese versions of DASH questionnaire have been proved to be valid and reliable for measurement of patients with upper extremity disorders, and they are Chinese Hong Kong translation [10] and Taiwan translation [11]. Both of the two translation versions were “traditional Chinese”. Compared with the “traditional Chinese”, the “simplified Chinese” is neologisms promulgated during the introduction of character simplification and Mandarin (the official Chinese language) to reduce interethnic and regional differences [12] and is prominently used in Mainland China and Singapore. The DASH has been translated into the simplified Chinese (Mainland) language but the validity and the reliability of the simplified Chinese (Mainland) version have never been investigated.

With regard to the discrepant cognitive process, as well as the differences on accessibility and quality of regional health care, this study attempted to translate the English version of DASH [4] into simplified Chinese, and to evaluate the reliability and validity.

## Methods

### Design

There were two phases included in this study. Firstly, the English version of the DASH was translated into simplified Chinese (Mainland), which was called cross-cultural adaptation. Secondly, the DASH was applied on patients with upper extremity problems to validate its reliability and validity. The study was performed according to the local ethical guidelines and was obtained from the patients.

### The DASH questionnaire

The DASH questionnaire consists mainly of a 30-item disability/symptom scale (DASH-D/S) concerning the health status of the patients during the preceding week [13]. These items involve the following: (1) inquiry of the difficulty degree in performing various physical activities due to the upper extremity problems (21 items); (2) the severity of the activity-related pain, symptoms of pain, tingling, stiffness, and weakness (5 items); and (3) the effects on work, activities, sleep, as well as on psychological impact (4 items).

Each of the DASH-D/S items has five alternative responses, with a score of 1 to 5 points. If at least 27 of the 30 items are completed, a score ranging from 0 to 100 can be computed as follows: [(sum of *n* responses/*n*) − 1] × 25, where *n* is the number of completed responses [14]. The higher the DASH score, the worse the disability would be.

### Translation and cultural adaptation of DASH

The English version of the DASH [4] was translated into simplified Chinese (Mainland) by two bilingual language officers whose native language was simplified Chinese (Mainland). Their translation versions were reviewed and discussed by each other, and a synthesis translation was produced with another language officer. An anesthesiologist and an academic professor whose first language is English were invited to translate this combined Chinese version back to English. This is a process of validity to make sure the translated version was accurate to the preliminary version. Both forward and back translators were unfamiliar and unaware of the concept of the DASH. An expert committee comprised of external orthopedic surgeons and physiotherapists was set up to evaluate the combined translation. Finally, the DASH simplified Chinese (Mainland) version (DASH-CHNPLAGH) was used to test patients.

### Setting and participants

To examine the psychometric properties and clinical application of the DASH-CHNPLAGH, a survey on those who underwent a previous surgery and were treated at the Department of Orthopaedic Surgery of the General Hospital of Chinese People’s Liberation Army (PLA) in China from 2010 to 2013 was conducted. The study excluded the patients (1) who were unable to complete the questionnaire independently due to language difficulties and cognitive impairment; (2) whose symptoms had changed between the first and second measurements; (3) whose disease were less than 2 months or who have pain from internal organs; and (4) whose age were less than 18 years old. The eligible patients were sent a package containing an invitation to participate, a consent form, a stamped return envelope, the adapted DASH-CHNPLAGH, as well as a previously validated Chinese (Mainland) version of the Short-Form (SF-36) Health Survey and a visual analogue scale (VAS) for pain measurement. The same package was sent again after 3 weeks. The SF-36 is a brief questionnaire that consists of eight dimensions of health, including physical functioning (PF), bodily pain (BP), general health (GH), role limitations due to physical problems (RP) and emotional problems (RE), vitality (VT), social functioning (SF), and mental health (MH) [15].

### Reliability

The Cronbach’s alpha coefficient was used to assess the internal consistency of the DASH-CHNPLAGH scale to other numerous versions. The test–retest reliability was evaluated by administering the DASH-CHNPLAGH questionnaire once again to the same patients 3 weeks later, with the intraclass coefficient (ICC) and Bland–Altman plot method [16].

### Validity

Convergent validity was evaluated by comparing the questionnaire DASH scores with relevant domains of the SF-36 questionnaire, including PF, RP, BP, GH, VT, SF, RE, MH, as well as physical component score (PCS) and mental component score (MCS). Correlation was assessed using the Pearson correlation coefficients (*R*).

### Statically analysis

The SPSS 21.0 (SPSS, Statistical Package for the Social Sciences) was used for all of the analysis, and the statistically significant was chosen at *P* < 0.05.

## Results

### Demographic and clinical data

A total of 300 patients (157 women and 143 men) with upper extremity problems who were aged between 18 and 76 years (mean age: 46.7 ± 29.4 year) and whose mother language is Chinese were invited to this study (Table 1). These patients underwent the mean disease duration of 17.3 (range: 2–180 months) months. Among them, 45 (15 %) had soft tissue injuries, 210 (70 %) had fractures, 26 (8.7 %) had nerve injury, 11 with multiple regions, 8 had dislocation. Twenty-six of them did not answer item 21 regarding sexual activity, and 13 patients did not respond to item 4 about preparing a simple meal.

**Table 1:** Demographic and clinical characteristics of patients

			Number of patients (%)
Sex			157 female (52.33)
Injured side	Left (%)		129 (43.00)
	Right (%)		153 (51.00)
	Bilateral (%)		18 (6.00)
Affected regions	Hand		44 (14.67)
	Wrist		86 (28.67)
	Forearm		23 (7.67)
	Elbow		61 (20.33)
	Brachium		7 (2.33)
	Shoulder		67 (32.33)
Diagnoses	Soft tissue injuries		45 (15.00)
		Frozen shoulder	17 (5.67)
		Sprain	10 (3.33)
		Tendinitis	7 (2.33)
		Trigger finger	5 (1.67)
		Cut tendon	6 (2.00)
	Fractures		210 (70.00)
		Hand fracture	35 (11.67)
		Wrist fracture	78 (26.00)
		Elbow fracture	54 (18.00)
		Shoulder fracture	43 (14.33)
	Nerve injury		26 (8.67)
		Brachial plexus	4 (1.33)
		Median nerve	7 (2.33)
		Ulnar nerve	6 (2.00)
		Radial nerve	9 (3.00)
	Multiple regions		11 (3.67)
	Dislocation		8 (2.67)

The mean DASH-CHNPLAGH score was 43.6 (range 20–92). Neither 0 nor 100 were recorded on these patients, which would represent the maximum health status score (ceiling) and the minimum health status score (floor), respectively.

### Reliability

Internal consistency was relatively high, with the Cronbach’s alpha coefficient of 0.96. The mean DASH-CHNPLAGH scores at the first and the second visit were 43.7 (range 18–90) and 45.4 (range 20–93), respectively. Test–retest reliability analysis gave an ICC of 0.94, indicating excellent reliability. We compared the internal consistency and the ICC with that reported in precious studies to detect if our results were more effective than that in other languages (Table 2). The results showed that, no matter for internal consistency and for ICC, the reliability value was relatively high in our study.

**Table 2:** Internal consistency of various DASH studies

Study	The area and language evaluated	Internal consistency (a)	Test–retest reliability (ICC)
Fayad et al. (2008) [6]	France (French)	0.96	0.95
Varjú1 et al. (2008) [26]	Hungary (Hungarian)	0.94	0.89
Lee et al. (2008) [20]	Korea (Korean)	0.94	0.91
Imaeda et al. (2005) [8]	Japan (Japanese)	0.96	0.82
Lee et al. (2004) [10]	Hong Kong (Cantonese Chinese)	0.94	0.77
Liang et al. (2004) [11]	Taiwan (traditional Chinese)	0.96	0.90
Offenbächer et al. (2003) [27]	German (Germany)	0.95	0.90
Padua et al. (2003) [25]	Italy (Italian)	0.90	0.89
Rosales et al. (2002) [28]	Spain (Spanish)	0.95	–
Beaton et al. (2001) [22]	Canada and USA (English)	–	0.96
Atroshi et al. (2000) [29]	Sweden (Swedish)	0.96	0.92
This study	China (Mandarin Chinese)	0.96	0.94

### Validity

The construct validity was evaluated by analyzing the relationship of the DASH-CHNPLAGH scores with the VAS and SF-36 using Pearson’s correlation coefficient (Table 3). All the SF-36 scores, including PF (*R* = −0.63, *P* < 0.001), RP (*R* = −0.43, *P* < 0.05), BP (*R* = −0.69, *P* < 0.001), GH (*R* = −0.43, *P* < 0.05), VT (*R* = −0.47, *P* < 0.05), SF (*R* = −0.57, *P* < 0.001), RE (*R* = −0.27, *P* < 0.05), MH (*R* = −0.34, *P* < 0.05), as well as the PCS (*R* = −0.71, *P* < 0.05) and the MCS (*R* = −0.37, *P* < 0.05) were significantly and negatively correlated with DASH-CHNPLAGH scores. The VAS was also correlated with DASH-CHNPLAGH scores significantly, although the correlation was positive.

**Table 3:** The correlations between DASH scores and SF-36

	Mean (SD)	Pearson’s correlation with DASH (R)
SF-36 physical functioning (PF)	47.2 ± 11.3	−0.63^**^
SF-36 role physical (RP)	33.4 ± 19.7	−0.43^*^
SF-36 bodily pain (BP)	45.1 ± 12.6	−0.69^**^
SF-36 general health (GH)	34.7 ± 11.2	−0.43^*^
SF-36 vitality (VT)	42.3 ± 8.3	−0.47^*^
SF-36 social functioning (SF)	72.8 ± 10.1	−0.57^**^
SF-36 role emotion (RE)	33.5 ± 20.4	−0.27^*^
SF-36 mental health (MH)	42.7 ± 11.7	−0.34^*^
SF-36 physical component score (PCS)	37.4 ± 15.6	−0.71^**^
SF-36 mental component score (MCS)	42.1 ± 17.1	−0.37^*^
VAS [0–10]	3.2 ± 1.13	0.58^**^

^*^Correlation is significant at *P* < 0.05; ^**^correlation is significant at *P* < 0.001

## Discussion

Upper extremity disorders are associated with much work disability cost and health care [17, 18]. It is therefore important to apply a standard, reliable, and valid measurement for detection of health changes related to the specific upper extremity diseases. Our results strongly demonstrated that the Chinese version of the DASH-CHNPLAGH scale can be used for accessing the upper extremity conditions for Chinese Mainland patients. The ICC and Cronbach’s alpha for the 30-item DASH-D/S of the DASH-CHNPLAGH was 0.94 and 0.96, respectively, which were relatively high compared with that reported in precious studies. DASH-CHNPLAGH also showed significant correlation with the SF-36 and the VAS, indicating the physical problem caused by musculoskeletal diseases is closely associated to physical activities.

The cross-cultural adaptation emphasizes two courses, the translation of questionnaire and the adaptation to idiom, cultural settings, and lifestyle [19]. Most of the DASH questionnaires are in English and are adapted to American culture settings. However, the DASH has been translated into several languages and is extensively used in many countries. During the translation process, many discrepancies may occur due to the linguistic and cultural differences in our study. Though the translations were corrected through the back translation process, other problems have been discovered in the process of resolving these discrepancies in China. That is because there was no corresponding simplified Chinese word in linguistic perspective. For example, the word “wash” is easy to translate to the Chinese word “Xi (洗)”, while in phrase “wash wall”, the word should be translated into “Shua (刷)”. Many items like this in the original version need to be clarified during translation. Furthermore, most Chinese Mainland people live in apartments without garden, so we changed the item concerning “gardening or doing yard-work” into “do housework or move the furniture” in Chinese. The item no. 4 “prepare a meal” seems to be ambiguous, so we substituted it into “make a normal meal”. For the no. 18 item, “recreational activities in which you take some force or impact through your arm, shoulder or hand (e.g., golf, hammering, tennis, etc.)”, however, there are few people in China who play golf; on the contrary, in traditional Chinese culture, the older people will take care of their grandchildren, so we added the item “hold baby (younger than a 2-year-old baby)”. For the item no. 19, we added the game “play ping-pang” because ping-pang is very popular in China.

After the questionnaires were taken back, we found that 26 of the subjects did not answer item 21 regarding sexual activity and 13 patients did not respond to item 4 about preparing a meal. The high rate of failure to answer the item about sexual activity may suggest that many people are unwilling to express their personal affairs regarding sexual activity to others. The reason may be explained by the deep-rooted traditional ideology in most of the Chinese individuals. Therefore, we recommend a more euphemistic expression with regard to sex activity if it should be used in the future DASH questionnaires. Though we have changed the item no. 4 “prepare a meal” into “make a normal meal”, still, 13 patients did not answer this question. Probably, these patients, especially the orderly males, had never prepared a meal. This phenomenon suggests that the Chinese society, like the other Asian countries (e.g., Korea), faces a gender role problem at home based on Confucianism [20].

There are various opinions about the test interval time, ranging from 2 successive days to 1- to 2-week duration [21, 22]. Perhaps a longer interval will present a better result considering the number of items and conditions of patients [20] Therefore, retesting of all patients was conducted after 3 weeks, after which the patients may not remember the content of the questionnaire that they answered the last time.

The values of Cronbach’s alpha of more than 0.7 indicate satisfactory correlation, of more than 0.8 indicate excellent correlation, and of more than 0.9 indicate excellent correlation. The Cronbach’s alpha for the 30-item DASH-D/S of the DASH-CHNPLAGH was 0.96, which was comparable to that reported by Beaton et al. (0.96) [22]. These results suggested that both of the DASH-CHNPLAGH and the original versions of DASH have excellent correlations among the items. The construct validity of the DASH-CHNPLAGH questionnaire was also evaluated by the VAS and SF-36. The VAS is a common subjective measurement scale for pain. It showed high correlation with DASH-CHNPLAGH, due to the close relationship between physical activities and the pain-related dysfunction. Other studies have reported correlation between the DASH and VAS of 0.748 [23], 0.42 [24], 0.442 at rest, and 0.555 during activity [20], 0.64 [6], which are similar to the result of 0.58 that we obtained. Thus, we believe that the assessment of a pain-related dysfunction should be useful in the questionnaire about pain severity caused by activity. SF-36 is another widely used instrument for measurements of quality life related to changes caused by disease or treatment. It has also been chosen to assess the construct validity of DASH by two component scores, PCS and MCS. Liang et al. [11] showed that the construct validity of the Taiwanese version of the DASH, BP, PF, and RP of the SF-36 subscales were significantly correlated with DASH. The Italian study also showed significant correlation with the PF of 0.41, BP of 0.51, GH of 0.36, VT of 0.35, SF of 0.64, and MH of 0.70 in the SF-36 [25]. All these results were similar to our data. In our study, the correlation of DASH-CHNPLAGH with PCS was higher than that with MCS, which was consistent with previous study [20], suggesting that the SF-36 is more suitable for the measurement of physical dysfunction.

A limitation in this study is that we did not analyze the responsiveness by using the standardized responsiveness mean and the effect size because we did not conduct the questionnaire survey before surgery. Further studies are necessary to evaluate the responsiveness of DASH-CHNPLAGH to uncover the longitudinal changes after the treatment of upper extremity disorders.

## Conclusion

In conclusion, though we have changed and modified the original version based on Chinese cultural settings, the reliability and validity of the DASH-CHNPLAGH were as good as the original DASH. We conclude that the cross-cultural adaptation is successful to minimize the linguistic and cultural discrepancies between English speaking countries and China, and the DASH-CHNPLAGH will be useful in assessing upper extremity disabilities of the patients whose first language is Chinese.
